# First record of *Urostylishubeiensis* Ren (Hemiptera, Heteroptera, Urostylididae) from Japan, with an illustrated key to the Japanese urostylidid species

**DOI:** 10.3897/BDJ.10.e83656

**Published:** 2022-04-18

**Authors:** Jun Souma, Yoshiaki Sakai, Tadashi Ishikawa

**Affiliations:** 1 Entomological Laboratory, Graduate School of Bioresource and Bioenvironmental Sciences, Kyushu University, Fukuoka, Japan Entomological Laboratory, Graduate School of Bioresource and Bioenvironmental Sciences, Kyushu University Fukuoka Japan; 2 Research Fellowship for Young Scientists (DC1), Japan Society for the Promotion of Science, Tokyo, Japan Research Fellowship for Young Scientists (DC1), Japan Society for the Promotion of Science Tokyo Japan; 3 Kuta, Izuhara-machi, Tsushima-shi, Nagasaki-ken, Japan Kuta Izuhara-machi, Tsushima-shi, Nagasaki-ken Japan; 4 Laboratory of Entomology, Faculty of Agriculture, Tokyo University of Agriculture, Atsugi-shi, Kanagawa, Japan Laboratory of Entomology, Faculty of Agriculture, Tokyo University of Agriculture Atsugi-shi, Kanagawa Japan

**Keywords:** Heteroptera, Urostylididae, *Urostylishubeiensis*, true bug, first record, illustrated key, Japan, Tsushima Island

## Abstract

**Background:**

Although the Japanese species of Urostylididae are of interest to not only heteropteran taxonomists, but also to the public, an illustrated key for all species of the family from the country is lacking. To date, the urostylidid species *Urostylishubeiensis* Ren, 1997, has been known to occur in China and Korea, but not in Japan.

**New information:**

*Urostylishubeiensis* is recorded from Japan for the first time and represents the easternmost occurrence of this species. In Japan, it inhabits the broad-leaved forest of Tsushima Island and was found on *Quercusacutissima* Carruth. (Fagaceae). An illustrated key to the species of Urostylididae occurring in Japan is provided.

## Introduction

The phytophagous family Urostylididae Dallas, 1851 (Hemiptera, Heteroptera) comprises over 170 species in 11 genera, which are mainly distributed in eastern and southern Asia (Rider 2006, Schuh and Weirauch 2020). In Japan, the fauna of Urostylididae has been known for more than 100 years, with five common species in two genera (*Urochela* Dallas, 1850 and *Urostylis* Westwood, 1837) and well-referenced biological information, such as their host plant affinities; thus, they have long been considered to be fully elucidated. In other words, *Uroc.luteovaria* (Distant, 1881) was described from Japan approximately 140 years ago and feeds on deciduous trees of Rosaceae; *Uroc.quadrinotata* (Reuter, 1881) was recorded from Japan for the first time approximately 110 years ago as *Uroc.jozankeana* Matsumura, 1913 and feeds on deciduous trees of Ulmaceae; and *Uros.annulicornis* Scott, 1874, *Uros.striicornis* Scott, 1874 and *Uros.westwoodii* Scott, 1874 were described from Japan approximately 150 years ago and feed on deciduous trees of *Quercus* spp. (Fagaceae) (cf. Scott 1874, Distant 1881, Matsumura 1913, Yang 1939, Yasunaga et al. 1993, Kanyukova and Marusik 2006, Ishikawa and Miyamoto 2012, Ishikawa 2016). Additionally, *Uroc.luteovaria* is known as a pest of apple, apricot, cherry, pear and sakura and *Uros.striicornis* and *Uros.westwoodii* are known pests of deciduous oaks (Japanese Society of Applied Entomology and Zoology 2006). Moreover, all five species have been frequently recorded in faunistic studies (Nagashima et al. 2015, Ito 2020, Okuda 2020 etc.) and numerous photographs have been posted on the Internet by the public (Wildlife Research Society 2016, Tsukiji 2022, amongst others). In conclusion, the Urostylididae are considered to be attracting attention from not only heteropteran taxonomists, but also by the general public in Japan. However, in previous studies dealing with the Japanese Urostylididae (e.g. Yasunaga et al. 1993, Ishikawa and Miyamoto 2012), no identification key illustrating the diagnostic characteristics of all five species was provided, which may hinder identification by the public. Therefore, the publication of an illustrated key to the urostylidid species from Japan will not only improve the accuracy of many future faunistic studies, but will also help the public accurately recognise these familiar true bugs.

For the past six years, the authors and colleagues have collected an indeterminate species of *Urostylis* from Tsushima Island, Japan. After careful morphological examination, we concluded that it represented the *Uros.hubeiensis* Ren, 1997, known to occur in China and Korea to date (Ren 1997, Kim et al. 2018, Zhou and Rédei 2018). Herein, we record *Uros.hubeiensis* from Japan for the first time, representing the easternmost occurrence of this species. In addition, we provide an illustrated key for the Japanese species of Urostylididae, including *Uros.hubeiensis*.

## Materials and methods

Dried specimens were used to observe the morphological characteristics. To examine the genitalia, the male terminalia were removed from the body after softening the specimens in hot water. The removed parts were immersed in a hot 15% potassium hydroxide (KOH) solution for 5 min and then soaked in 99% ethanol for further observation. Male genitalia were preserved in small polyethylene vials containing 50% glycerine and mounted on a pin with the respective specimens. Morphological characteristics were observed and measured using a stereoscopic microscope (SZ60; Olympus, Tokyo, Japan), equipped with an ocular grid. The specimens were photographed using a digital microscope (Dino-Lite Premier M; Opto Science, Tokyo, Japan) and a compact digital camera (Tough TG-6; Olympus, Tokyo, Japan) and image stacks were processed using Adobe Photoshop 2021 ver. 22.5.1 (Adobe Inc., California, U.S.A.) when using a digital microscope. Morphological terms were generally assigned in accordance with Zhou and Rédei (2018).

All specimens of *Urostylishubeiensis* used in the present study were deposited in the Laboratory of Entomology, Faculty of Agriculture, Tokyo University of Agriculture, Kanagawa, Japan (TUA). Specimens of the Japanese Urostylididae that were used for creating the identification key and comparison with *Uros.hubeiensis* were deposited in the Entomological Laboratory, Faculty of Agriculture, Kyushu University, Fukuoka, Japan (ELKU) and TUA.

Distribution records of the species were mapped using SimpleMappr software (Shorthouse 2010). Geographical coordinates were obtained using the Google Maps software. The map was edited using Adobe Photoshop 2021 ver.22.5.1. The scientific names of the host plants were assigned in accordance with Yonekura and Kajita (2022).

## Taxon treatments

### Urostylis hubeiensis

Ren, 1997

CB8F7B65-7927-5E98-9C34-32967DBFCF09

Urostylis hubeiensis Ren, 1997 - Ren 1997 [58], new species, description and figures; Rider 2006 [113], catalogue and distribution; Zhou and Rédei 2018: 145, diagnosis, figures and distribution.
Urostylis koreana Kim & Jung, 2018 - Kim et al. 2018 [446], new species, description and figures; Zhou and Rédei 2018: 145, synonymised with *Urostylishubeiensis*.

#### Materials

- **Type status:** Other material. **Occurrence:** recordedBy: Yoshiaki Sakai; individualCount: 1; sex: male; lifeStage: adult; **Taxon:** scientificName: *Urostylishubeiensis* Ren, 1997; namePublishedIn: 1997; kingdom: Animalia; phylum: Arthropoda; class: Insecta; order: Hemiptera; family: Urostylididae; genus: Urostylis; specificEpithet: *hubeiensis*; scientificNameAuthorship: Ren; **Location:** island: Tsushima Island; country: Japan; stateProvince: Nagasaki; municipality: Toyotama-machi; locality: Nii; decimalLatitude: 34.401000; decimalLongitude: 129.326000; geodeticDatum: WGS84; **Identification:** identifiedBy: Jun Souma; Tadashi Ishikawa; dateIdentified: 2022; **Event:** samplingProtocol: none specified; eventDate: 22/11/2016; **Record Level:** institutionCode: TUA; basisOfRecord: PreservedSpecimen
- **Type status:** Other material. **Occurrence:** recordedBy: Yoshiaki Sakai; individualCount: 1; sex: female; lifeStage: adult; **Taxon:** scientificName: *Urostylishubeiensis* Ren, 1997; namePublishedIn: 1997; kingdom: Animalia; phylum: Arthropoda; class: Insecta; order: Hemiptera; family: Urostylididae; genus: Urostylis; specificEpithet: *hubeiensis*; scientificNameAuthorship: Ren; **Location:** island: Tsushima Island; country: Japan; stateProvince: Nagasaki; municipality: Toyotama-machi; locality: Nii; decimalLatitude: 34.401000; decimalLongitude: 129.326000; geodeticDatum: WGS84; **Identification:** identifiedBy: Jun Souma; Tadashi Ishikawa; dateIdentified: 2022; **Event:** samplingProtocol: none specified; eventDate: 22/11/2016; **Record Level:** institutionCode: TUA; basisOfRecord: PreservedSpecimen
- **Type status:** Other material. **Occurrence:** recordedBy: Yoshiaki Sakai; individualCount: 2; sex: female; lifeStage: adult; **Taxon:** scientificName: *Urostylishubeiensis* Ren, 1997; namePublishedIn: 1997; kingdom: Animalia; phylum: Arthropoda; class: Insecta; order: Hemiptera; family: Urostylididae; genus: Urostylis; specificEpithet: *hubeiensis*; scientificNameAuthorship: Ren; **Location:** island: Tsushima Island; country: Japan; stateProvince: Nagasaki; municipality: Toyotama-machi; locality: Nii, Matsunbara-shizen-kôen; decimalLatitude: 34.404940; decimalLongitude: 129.332420; geodeticDatum: WGS84; **Identification:** identifiedBy: Jun Souma; Tadashi Ishikawa; dateIdentified: 2022; **Event:** samplingProtocol: none specified; eventDate: 05/09/2017; **Record Level:** institutionCode: TUA; basisOfRecord: PreservedSpecimen
- **Type status:** Other material. **Occurrence:** recordedBy: Tadashi Ishikawa; individualCount: 2; sex: female; lifeStage: adult; **Taxon:** scientificName: *Urostylishubeiensis* Ren, 1997; namePublishedIn: 1997; kingdom: Animalia; phylum: Arthropoda; class: Insecta; order: Hemiptera; family: Urostylididae; genus: Urostylis; specificEpithet: *hubeiensis*; scientificNameAuthorship: Ren; **Location:** island: Tsushima Island; country: Japan; stateProvince: Nagasaki; municipality: Toyotama-machi; locality: Nii, Matsunbara-shizen-kôen; decimalLatitude: 34.404940; decimalLongitude: 129.332420; geodeticDatum: WGS84; **Identification:** identifiedBy: Jun Souma; Tadashi Ishikawa; dateIdentified: 2022; **Event:** samplingProtocol: none specified; eventDate: 05/09/2017; **Record Level:** institutionCode: TUA; basisOfRecord: PreservedSpecimen
- **Type status:** Other material. **Occurrence:** recordedBy: Jun Souma; individualCount: 1; sex: female; lifeStage: adult; **Taxon:** scientificName: *Urostylishubeiensis* Ren, 1997; namePublishedIn: 1997; kingdom: Animalia; phylum: Arthropoda; class: Insecta; order: Hemiptera; family: Urostylididae; genus: Urostylis; specificEpithet: *hubeiensis*; scientificNameAuthorship: Ren; **Location:** island: Tsushima Island; country: Japan; stateProvince: Nagasaki; municipality: Toyotama-machi; locality: Nii, Matsunbara-shizen-kôen; decimalLatitude: 34.404940; decimalLongitude: 129.332420; geodeticDatum: WGS84; **Identification:** identifiedBy: Jun Souma; Tadashi Ishikawa; dateIdentified: 2022; **Event:** samplingProtocol: none specified; eventDate: 06/09/2017; **Record Level:** institutionCode: TUA; basisOfRecord: PreservedSpecimen
- **Type status:** Other material. **Occurrence:** recordedBy: Yoshiaki Sakai; individualCount: 4; sex: male; lifeStage: adult; **Taxon:** scientificName: *Urostylishubeiensis* Ren, 1997; namePublishedIn: 1997; kingdom: Animalia; phylum: Arthropoda; class: Insecta; order: Hemiptera; family: Urostylididae; genus: Urostylis; specificEpithet: *hubeiensis*; scientificNameAuthorship: Ren; **Location:** island: Tsushima Island; country: Japan; stateProvince: Nagasaki; municipality: Toyotama-machi; locality: Nii, Matsunbara-shizen-kôen; decimalLatitude: 34.404940; decimalLongitude: 129.332420; geodeticDatum: WGS84; **Identification:** identifiedBy: Jun Souma; Tadashi Ishikawa; dateIdentified: 2022; **Event:** samplingProtocol: none specified; eventDate: 17/11/2017; **Record Level:** institutionCode: TUA; basisOfRecord: PreservedSpecimen
- **Type status:** Other material. **Occurrence:** recordedBy: Yoshiaki Sakai; individualCount: 2; sex: female; lifeStage: adult; **Taxon:** scientificName: *Urostylishubeiensis* Ren, 1997; namePublishedIn: 1997; kingdom: Animalia; phylum: Arthropoda; class: Insecta; order: Hemiptera; family: Urostylididae; genus: Urostylis; specificEpithet: *hubeiensis*; scientificNameAuthorship: Ren; **Location:** island: Tsushima Island; country: Japan; stateProvince: Nagasaki; municipality: Toyotama-machi; locality: Nii, Matsunbara-shizen-kôen; decimalLatitude: 34.404940; decimalLongitude: 129.332420; geodeticDatum: WGS84; **Identification:** identifiedBy: Jun Souma; Tadashi Ishikawa; dateIdentified: 2022; **Event:** samplingProtocol: none specified; eventDate: 17/11/2017; **Record Level:** institutionCode: TUA; basisOfRecord: PreservedSpecimen
- **Type status:** Other material. **Occurrence:** recordedBy: Yoshiaki Sakai; individualCount: 1; sex: female; lifeStage: adult; **Taxon:** scientificName: *Urostylishubeiensis* Ren, 1997; namePublishedIn: 1997; kingdom: Animalia; phylum: Arthropoda; class: Insecta; order: Hemiptera; family: Urostylididae; genus: Urostylis; specificEpithet: *hubeiensis*; scientificNameAuthorship: Ren; **Location:** island: Tsushima Island; country: Japan; stateProvince: Nagasaki; municipality: Mine-machi; locality: Saka; decimalLatitude: 34.464881; decimalLongitude: 129.372242; geodeticDatum: WGS84; **Identification:** identifiedBy: Jun Souma; Tadashi Ishikawa; dateIdentified: 2022; **Event:** samplingProtocol: none specified; eventDate: 27/05/2018; **Record Level:** institutionCode: TUA; basisOfRecord: PreservedSpecimen
- **Type status:** Other material. **Occurrence:** recordedBy: Yoshiaki Sakai; individualCount: 2; sex: male; lifeStage: adult; **Taxon:** scientificName: *Urostylishubeiensis* Ren, 1997; namePublishedIn: 1997; kingdom: Animalia; phylum: Arthropoda; class: Insecta; order: Hemiptera; family: Urostylididae; genus: Urostylis; specificEpithet: *hubeiensis*; scientificNameAuthorship: Ren; **Location:** island: Tsushima Island; country: Japan; stateProvince: Nagasaki; municipality: Mine-machi; locality: Saka; decimalLatitude: 34.464881; decimalLongitude: 129.372242; geodeticDatum: WGS84; **Identification:** identifiedBy: Jun Souma; Tadashi Ishikawa; dateIdentified: 2022; **Event:** samplingProtocol: none specified; eventDate: 02/06/2018; **Record Level:** institutionCode: TUA; basisOfRecord: PreservedSpecimen
- **Type status:** Other material. **Occurrence:** recordedBy: Yoshiaki Sakai; individualCount: 1; sex: male; lifeStage: adult; **Taxon:** scientificName: *Urostylishubeiensis* Ren, 1997; namePublishedIn: 1997; kingdom: Animalia; phylum: Arthropoda; class: Insecta; order: Hemiptera; family: Urostylididae; genus: Urostylis; specificEpithet: *hubeiensis*; scientificNameAuthorship: Ren; **Location:** island: Tsushima Island; country: Japan; stateProvince: Nagasaki; municipality: Mine-machi; locality: Saka; decimalLatitude: 34.464881; decimalLongitude: 129.372242; geodeticDatum: WGS84; **Identification:** identifiedBy: Jun Souma; Tadashi Ishikawa; dateIdentified: 2022; **Event:** samplingProtocol: none specified; eventDate: 01/06/2019; **Record Level:** institutionCode: TUA; basisOfRecord: PreservedSpecimen
- **Type status:** Other material. **Occurrence:** recordedBy: Shusuke Shimamoto; individualCount: 2; sex: male; lifeStage: adult; **Taxon:** scientificName: *Urostylishubeiensis* Ren, 1997; namePublishedIn: 1997; kingdom: Animalia; phylum: Arthropoda; class: Insecta; order: Hemiptera; family: Urostylididae; genus: Urostylis; specificEpithet: *hubeiensis*; scientificNameAuthorship: Ren; **Location:** island: Tsushima Island; country: Japan; stateProvince: Nagasaki; municipality: Toyotama-machi; locality: Nii, Matsunbara Shinrin Koen; decimalLatitude: 34.404940; decimalLongitude: 129.332420; geodeticDatum: WGS84; **Identification:** identifiedBy: Jun Souma; Tadashi Ishikawa; dateIdentified: 2022; **Event:** samplingProtocol: none specified; eventDate: 08/06/2019; **Record Level:** institutionCode: TUA; basisOfRecord: PreservedSpecimen
- **Type status:** Other material. **Occurrence:** recordedBy: Shusuke Shimamoto; individualCount: 3; sex: female; lifeStage: adult; **Taxon:** scientificName: *Urostylishubeiensis* Ren, 1997; namePublishedIn: 1997; kingdom: Animalia; phylum: Arthropoda; class: Insecta; order: Hemiptera; family: Urostylididae; genus: Urostylis; specificEpithet: *hubeiensis*; scientificNameAuthorship: Ren; **Location:** island: Tsushima Island; country: Japan; stateProvince: Nagasaki; municipality: Toyotama-machi; locality: Nii, Matsunbara Shinrin Koen; decimalLatitude: 34.404940; decimalLongitude: 129.332420; geodeticDatum: WGS84; **Identification:** identifiedBy: Jun Souma; Tadashi Ishikawa; dateIdentified: 2022; **Event:** samplingProtocol: none specified; eventDate: 08/06/2019; **Record Level:** institutionCode: TUA; basisOfRecord: PreservedSpecimen
- **Type status:** Other material. **Occurrence:** recordedBy: Shusuke Shimamoto; individualCount: 1; sex: male; lifeStage: adult; **Taxon:** scientificName: *Urostylishubeiensis* Ren, 1997; namePublishedIn: 1997; kingdom: Animalia; phylum: Arthropoda; class: Insecta; order: Hemiptera; family: Urostylididae; genus: Urostylis; specificEpithet: *hubeiensis*; scientificNameAuthorship: Ren; **Location:** island: Tsushima Island; country: Japan; stateProvince: Nagasaki; municipality: Kamitsushima-machi; locality: Kawachi, Yuishiyama Shinrin Koen; decimalLatitude: 34.673087; decimalLongitude: 129.421658; geodeticDatum: WGS84; **Identification:** identifiedBy: Jun Souma; Tadashi Ishikawa; dateIdentified: 2022; **Event:** samplingProtocol: none specified; eventDate: 13/06/2019; **Record Level:** institutionCode: TUA; basisOfRecord: PreservedSpecimen
- **Type status:** Other material. **Occurrence:** recordedBy: Shusuke Shimamoto; individualCount: 1; sex: female; lifeStage: adult; **Taxon:** scientificName: *Urostylishubeiensis* Ren, 1997; namePublishedIn: 1997; kingdom: Animalia; phylum: Arthropoda; class: Insecta; order: Hemiptera; family: Urostylididae; genus: Urostylis; specificEpithet: *hubeiensis*; scientificNameAuthorship: Ren; **Location:** island: Tsushima Island; country: Japan; stateProvince: Nagasaki; municipality: Kamitsushima-machi; locality: Kawachi, Yuishiyama Shinrin Koen; decimalLatitude: 34.673087; decimalLongitude: 129.421658; geodeticDatum: WGS84; **Identification:** identifiedBy: Jun Souma; Tadashi Ishikawa; dateIdentified: 2022; **Event:** samplingProtocol: none specified; eventDate: 13/06/2019; **Record Level:** institutionCode: TUA; basisOfRecord: PreservedSpecimen

#### Diagnosis

**Figure 1.**
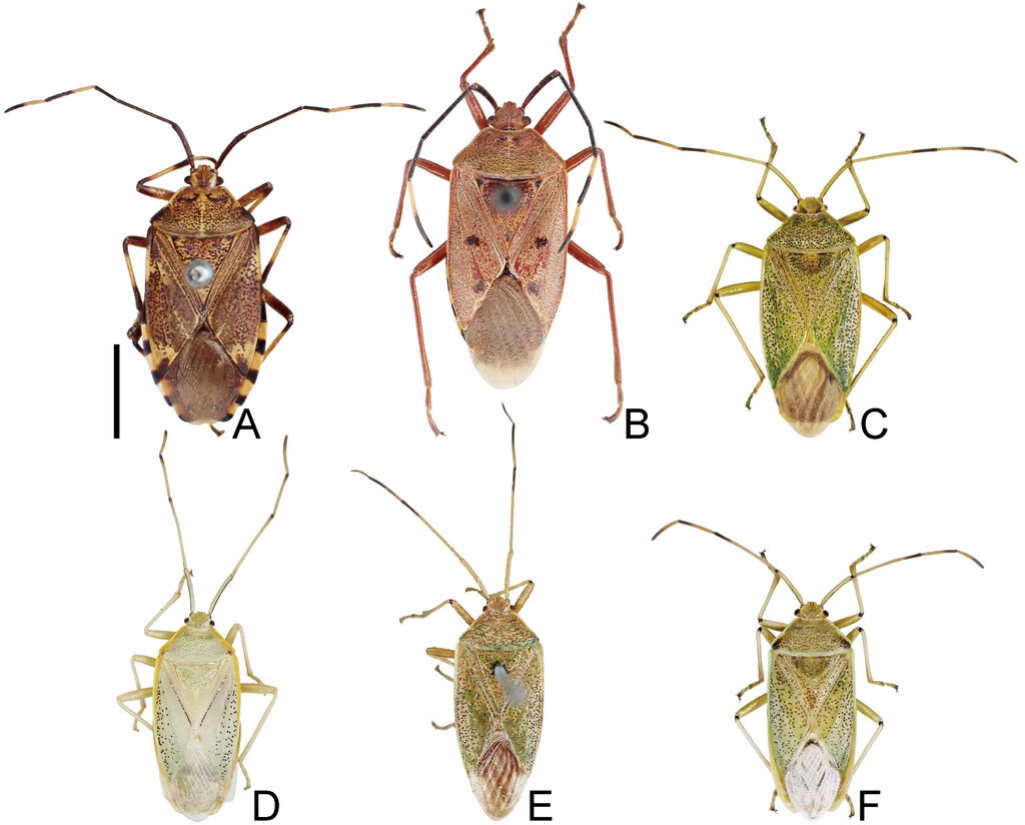
Dorsal habitus of six species of Urostylididae from Japan. Scale bar: 5.0 mm. **A** *Urochelaluteovaria*; **B** *Urochelaquadrinotata*; **C** *Urostylisannulicornis*; **D** *Urostylishubeiensis*; **E** *Urostylisstriicornis*; **F** *Urostyliswestwoodii*.

**Figure 2.**
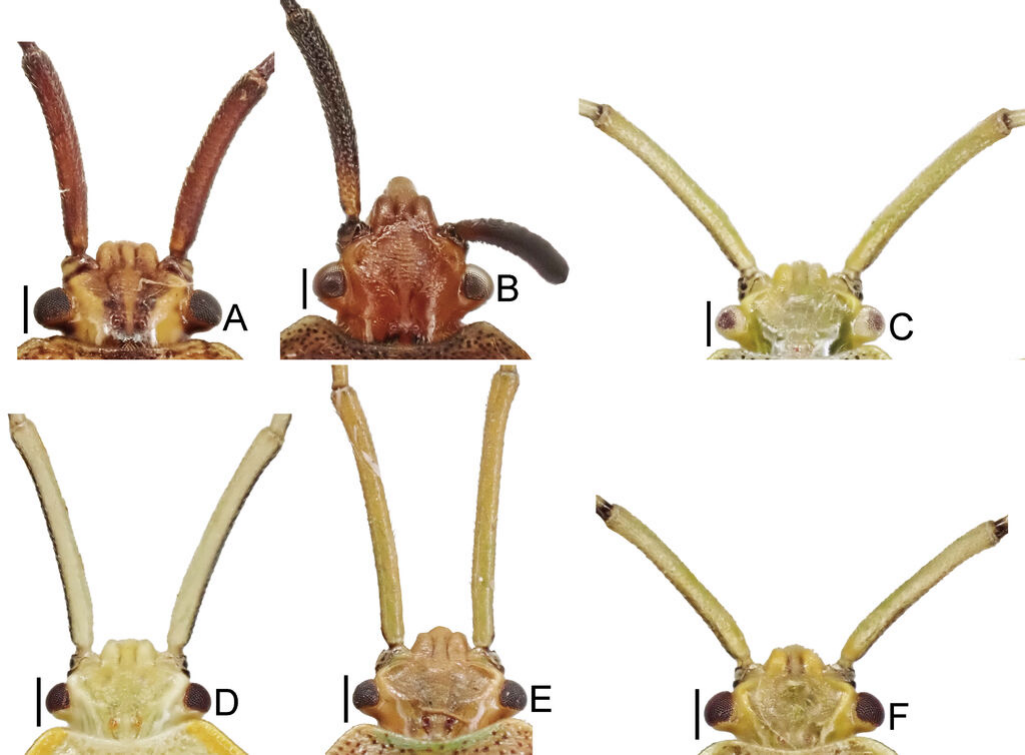
Head and scape of six species of Urostylididae from Japan, dorsal view. Scale bars: 0.5 mm. **A** *Urochelaluteovaria*; **B** *Urochelaquadrinotata*; **C** *Urostylisannulicornis*; **D** *Urostylishubeiensis*; **E** *Urostylisstriicornis*; **F** *Urostyliswestwoodii*.

**Figure 3.**
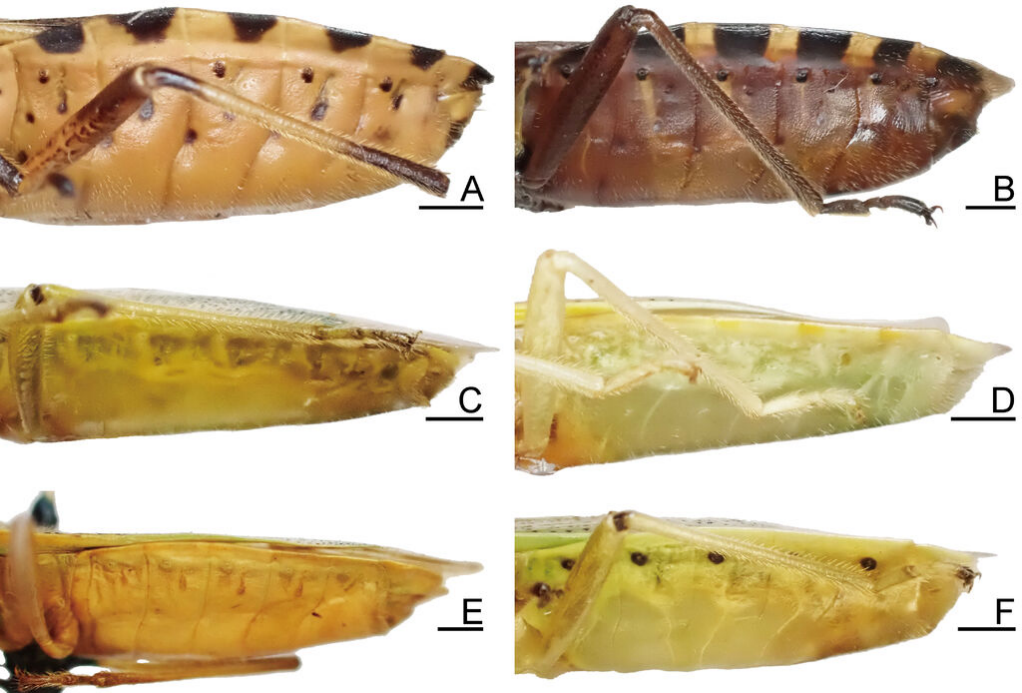
Female abdomen of six species of Urostylididae from Japan, lateral view. Scale bars: 1.0 mm. **A** *Urochelaluteovaria*; **B** *Urochelaquadrinotata*; **C** *Urostylisannulicornis*; **D** *Urostylishubeiensis*; **E** *Urostylisstriicornis*; **F** *Urostyliswestwoodii*.

**Figure 4.**
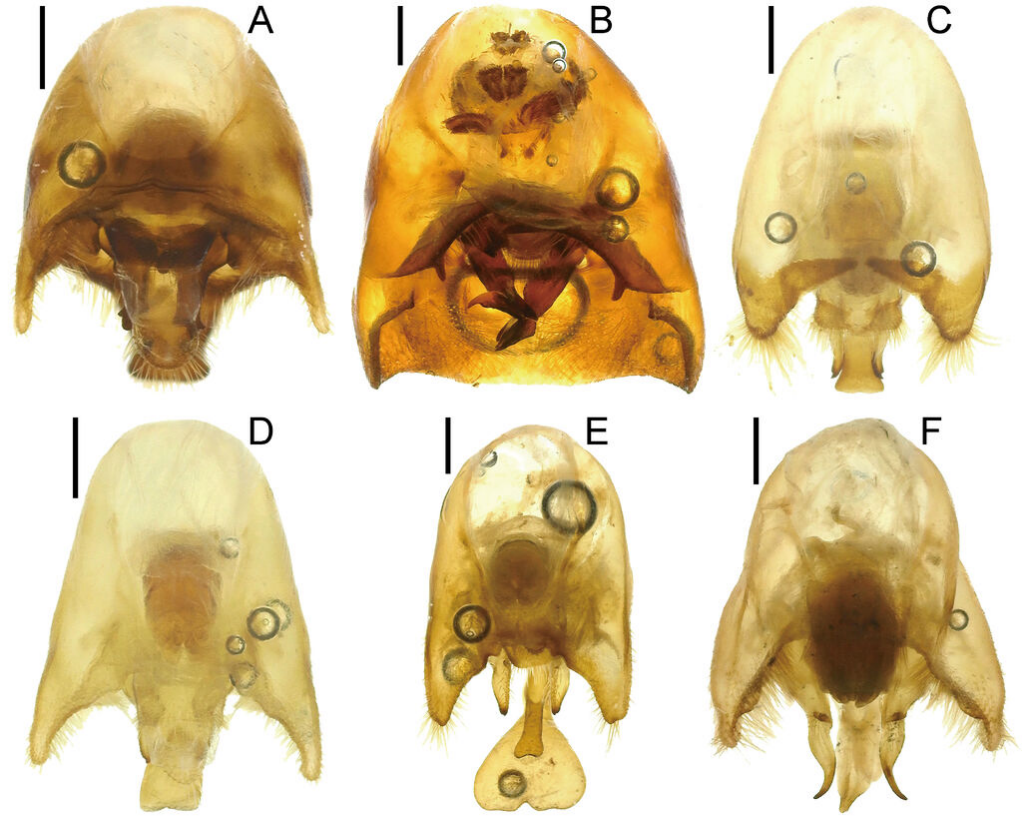
Genital capsule of six species of Urostylididae from Japan, dorsal view. Scale bars: 0.5 mm. **A** *Urochelaluteovaria*; **B** *Urochelaquadrinotata*; **C** *Urostylisannulicornis*; **D** *Urostylishubeiensis*; **E** *Urostylisstriicornis*; **F** *Urostyliswestwoodii*.

**Figure 5.**
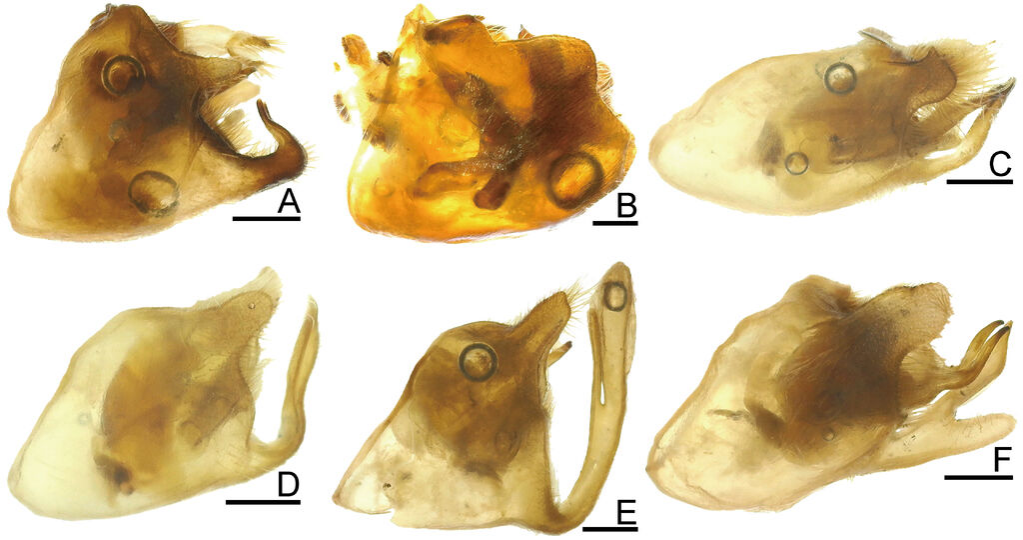
Genital capsule of six species of Urostylididae from Japan, lateral view. Scale bars: 0.5 mm. **A** *Urochelaluteovaria*; **B** *Urochelaquadrinotata*; **C** *Urostylisannulicornis*; **D** *Urostylishubeiensis*; **E** *Urostylisstriicornis*; **F** *Urostyliswestwoodii*.

**Figure 6.**
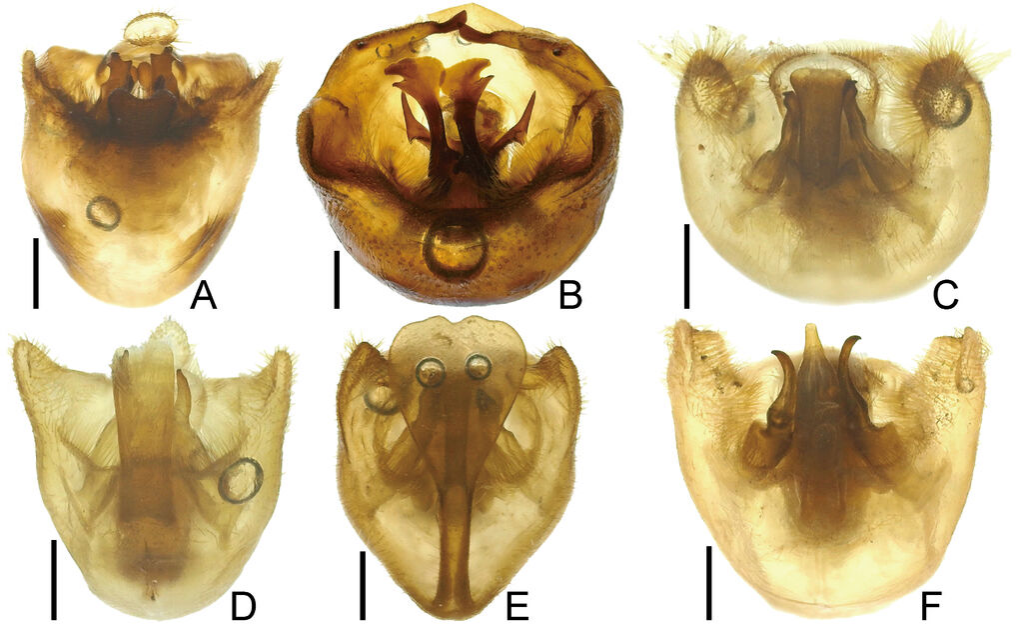
Genital capsule of six species of Urostylididae from Japan, caudal view. Scale bars: 0.5 mm. **A** *Urochelaluteovaria*; **B** *Urochelaquadrinotata*; **C** *Urostylisannulicornis*; **D** *Urostylishubeiensis*; **E** *Urostylisstriicornis*; **F** *Urostyliswestwoodii*.

**Figure 7.**
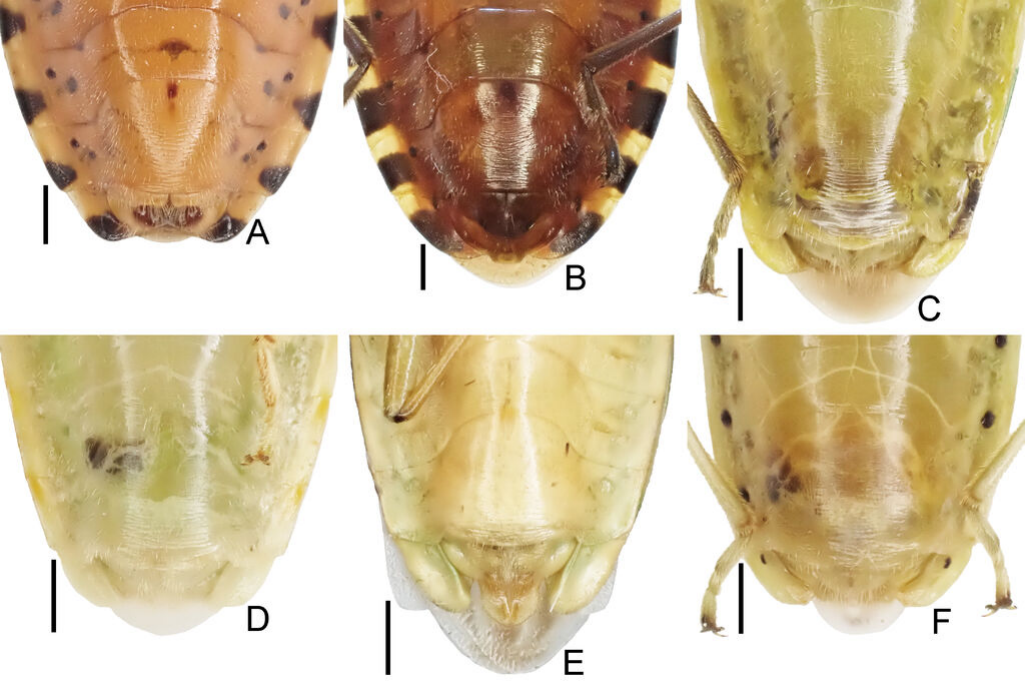
Female terminalia of six species of Urostylididae from Japan, ventral view. Scale bars: 1.0 mm. **A** *Urochelaluteovaria*; **B** *Urochelaquadrinotata*; **C** *Urostylisannulicornis*; **D** *Urostylishubeiensis*; **E** *Urostylisstriicornis*; **F** *Urostyliswestwoodii*.

**Figure 8.**
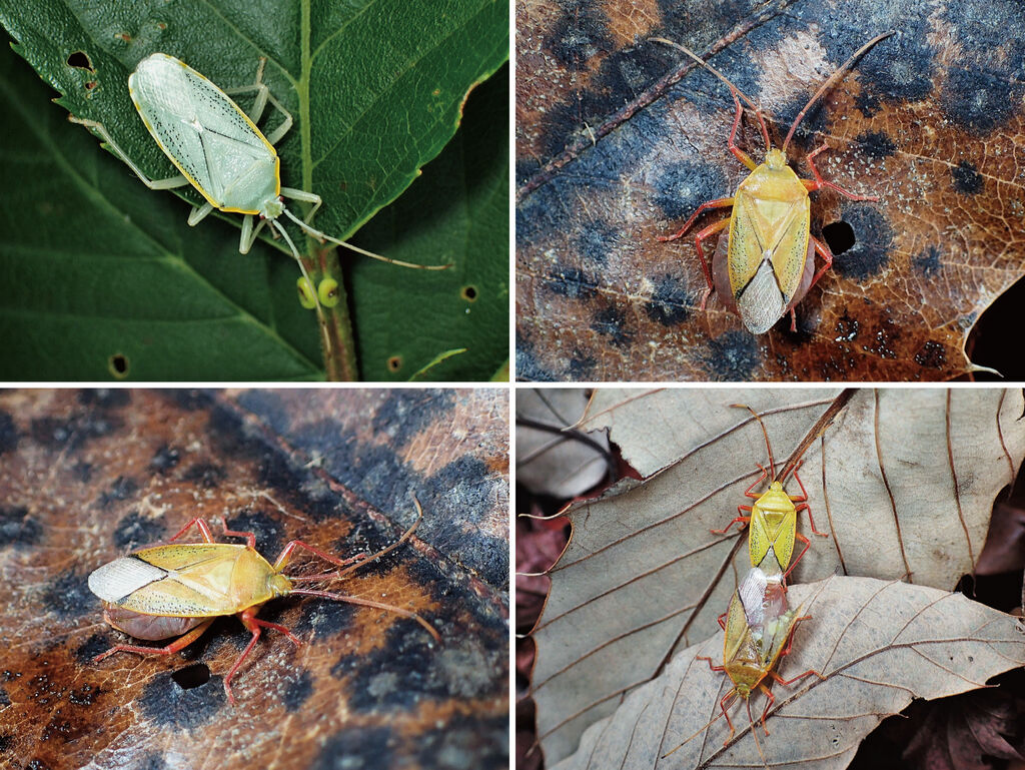
Habitus images of live individuals of *Urostylishubeiensis* from Tsushima Island, Japan.

*Urostylishubeiensis* can be distinguished from other species of the genus using a combination of the following characteristics (cf. Kim et al. 2018, Zhou and Rédei 2018): dorsum pale, mostly concolorously punctate (Fig. 1D and Fig. 8); scape (antennal segment I) with a black longitudinal fascia on lateral surface (Fig. 2D); scutellum with pale punctures entirely; forewing decorated with large, coarse, scattered black punctures in area enclosed by medial furrow and Cu (cubital) vein (relatively sparse between Cu and R+M (radiomedial) veins, denser between R+M and medial furrow); corium with a series of rather regularly arranged, closely placed black punctures forming a straight line along claval furrow; abdomen with pale spiracles (Fig. 3D); genital capsule of male with a pair of short and blunt dorsolateral processes and a single apically excised ventromedian process (Fig. 4D, Fig. 5D and Fig. 6D); posterior margin of genital capsule below dorsolateral process broadly convex, enclosing a narrow, U-shaped interspace with anterior margin of ventromedian process in lateral view; ventromedian process directed nearly dorsad, with apex approximately reaching level of dorsolateral processes in lateral view, with lateral margins subparallel-sided or slightly concave in caudal view; ventrite VII of female not protruding posteriad below ovipositor, leaving valvifers VIII fully exposed (Fig. 7D); laterotergite VIII of female reaching a level of laterotergite IX ventrally; laterotergite IX of female longer, distinctly surpassing posterior extremities of valvifers VIII both in ventral, but particularly in posteroventral views; and posteromesal portion of laterotergite IX surpassing its posterolateral portion posteriorly.

#### Distribution

**Figure 9.**
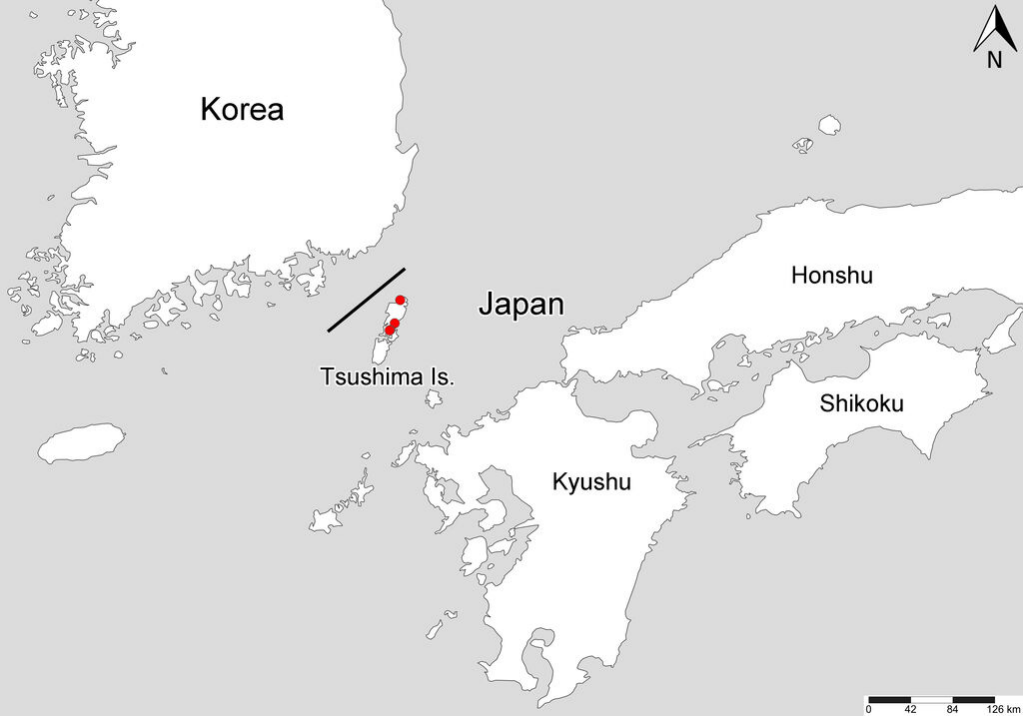
Collection sites of *Urostylishubeiensis* from Tsushima Island, Japan.

Japan (Tsushima Island) (Fig. 9), China (Ren 1997, Zhou and Rédei 2018), Korea (Kim et al. 2018, Zhou and Rédei 2018).

The records of *Urostylishubeiensis* from Tsushima Island represent the easternmost occurrence of this species.

#### Biology

*Urostylishubeiensis* was collected from *Quercusacutissima* Carruth. (Fagaceae) in Japan, suggesting that this deciduous fagaceous tree is a host plant for this species. In other distribution areas, the host plant of this urostylidid species is unknown. *Uros.hubeiensis* is found in broad-leaved forests in Japan. Its habitat is unknown in China, but it is found regularly in forest of *Q.acutissima* in Korea (M. Roca-Cusachs, pers. comm. 2022). In Korea, *Uros.hubeiensis* were attracted to artificial light (Kim et al. 2018).

Adults were collected in May, June, September and November in Japan and from May to September in other distribution areas (Ren 1997, Kim et al. 2018, Zhou and Rédei 2018). The nymph and overwintering stages are unknown both in Japan and in other distribution areas.

#### Taxon discussion

The above-recorded specimens matched the photographs of the holotype (Zhou and Rédei 2018) and descriptions (Ren 1997, Kim et al. 2018, Zhou and Rédei 2018) of *Urostylishubeiensis* described from China in terms of their morphological characteristics. Moreover, the Japanese specimens were identified as *Uros.koreana* [= *Uros.hubeiensis*] using a key for the Korean species of *Urostylis* (Kim et al. 2018). In conclusion, we identified the Japanese specimens as *Uros.hubeiensis*.

## Checklists

### Checklist of the species of Urostylididae ocurring in Japan

#### Urochela luteovaria

(Distant, 1881)

616BF66F-E0CE-5435-A47D-182192A0E7CB

##### Distribution

China, Japan (Hokkaido, Honshu, Shikoku, Kyushu), Korea (Distant 1881, Rider 2006, Ishikawa and Miyamoto 2012, Ishikawa 2016).

#### Urochela quadrinotata

(Reuter, 1881)

F9C86734-E0E6-50CE-842A-B426C448AD29

##### Distribution

China, Japan (Hokkaido, Honshu, Shikoku, Kyushu), Korea, Russia (Matsumura 1913, Yang 1939, Rider 2006, Ishikawa and Miyamoto 2012, Ishikawa 2016).

#### Urostylis annulicornis

Scott, 1874

89F83B77-2D39-5A49-9065-FAD50EFE7D20

##### Distribution

China, Japan (Kunashiri Island, Hokkaido, Honshu, Shikoku, Kyushu), Korea, Russia (Scott 1874, Kanyukova and Marusik 2006, Rider 2006, Ishikawa and Miyamoto 2012, Ishikawa 2016).

#### Urostylis hubeiensis

Ren, 1997

9AAF865A-2F6E-5A1E-B816-56CDA83D8946

##### Distribution

China, Japan (Tsushima Island), Korea (Ren 1997, Kim et al. 2018, Zhou and Rédei 2018, present study).

#### Urostylis striicornis

Scott, 1874

6F9F61D4-B8E6-5BF7-8A3D-3613DCB19FCE

##### Distribution

China, Japan (Hokkaido, Honshu, Sado Island, Shikoku, Kyushu, Tsushima Island), Korea, Russia (Scott 1874, Rider 2006, Ishikawa and Miyamoto 2012, Ishikawa 2016).

#### Urostylis westwoodii

Scott, 1874

D839BEFE-0323-5851-9788-9A6225482AD4

##### Distribution

China, Japan (Honshu, Shikoku, Kyushu, Tsushima Island), Korea (Scott 1874, Rider 2006, Ishikawa and Miyamoto 2012, Ishikawa 2016).

## Identification Keys

### Key to the species of Urostylididae occurring in Japan

**Table:** 

1	Dorsum brown or reddish-brown (Fig. 1A and B); scape (antennal segment I) shorter, less than 2.0 times as long as head (Fig. 2A and B); laterotergites III–VII with a single black spot (Fig. 3A, B and Fig. 7A and B)	2
–	Dorsum pale or green (Fig. 1C–F and Fig. 8); scape longer, more than 2.0 times as long as head (Fig. 2C–F); laterotergites III–VII without black spot (Fig. 3C–F and Fig. 7C–F)	3
2	Corium of forewing without black spot (Fig. 1A); dorsolateral and ventromedian processes of genital capsule well-developed (Fig. 4A, Fig. 5A and Fig. 6A)	*Urochelaluteovaria* (Distant, 1881)
–	Corium of forewing with two black spots (Fig. 1B); dorsolateral and ventromedian processes of genital capsule undeveloped (Fig. 4B, Fig. 5B and Fig. 6B)	*Urochelaquadrinotata* (Reuter, 1881)
3	Abdomen with black spiracles (Fig. 3F and Fig. 7F); ventromedian process of genital capsule narrowed apically (Fig. 4F, Fig. 5F and Fig. 6F)	*Urostyliswestwoodii* Scott, 1874
–	Abdomen with pale spiracles (Fig. 3C–E and Fig. 7C–E); ventromedian process of genital capsule not narrowed apically (Fig. 4C–E, Fig. 5C–E and Fig. 6C–E)	4
4	Dorsum pale (Fig. 1D and Fig. 8); scutellum with pale punctures entirely; corium of forewing with black punctures partly	*Urostylishubeiensis* Ren, 1997
–	Dorsum green (Fig. 1C and E); scutellum with black punctures entirely; corium of forewing with black punctures entirely	5
5	Ventromedian process of genital capsule not widened apically (Fig. 4C, Fig. 5C and 6C); laterotergite IX not elongated, not protruding posteriad (Fig. 7C)	*Urostylisannulicornis* Scott, 1874
–	Ventromedian process of genital capsule widened apically (Fig. 4E, Fig. 5E and Fig. 6E); laterotergite IX elongated, protruding posteriad (Fig. 7E)	*Urostylisstriicornis* Scott, 1874

## Supplementary Material

XML Treatment for Urostylis hubeiensis

XML Treatment for Urochela luteovaria

XML Treatment for Urochela quadrinotata

XML Treatment for Urostylis annulicornis

XML Treatment for Urostylis hubeiensis

XML Treatment for Urostylis striicornis

XML Treatment for Urostylis westwoodii
